# Supplementary material for: Harnessing the central dogma for stringent multi-level control of gene expression
Source: Nat Commun. 2021 Mar 19;12:1738. doi: 10.1038/s41467-021-21995-7 (PMC7979795; doi:10.1038/s41467-021-21995-7)
Supplement: Supplementary file 4 — Reporting Summary [file 41467_2021_21995_MOESM4_ESM.pdf]

nature research

Corresponding author(s): Thomas Gorochowski

Last updated by author(s): Feb 17, 2021

Reporting Summary

Nature Research wishes to improve the reproducibility of the work that we publish. This form provides structure for consistency and transparency in reporting. For further information on Nature Research policies, see our Editorial Policies and the Editorial Policy Checklist.

Statistics

For all statistical analyses, confirm that the following items are present in the figure legend, table legend, main text, or Methods section.

n/a	Confirmed	
☐	☒	The exact sample size (*n*) for each experimental group/condition, given as a discrete number and unit of measurement
☐	☒	A statement on whether measurements were taken from distinct samples or whether the same sample was measured repeatedly
☒	☐	The statistical test(s) used AND whether they are one- or two-sided<br>*Only common tests should be described solely by name; describe more complex techniques in the Methods section.*
☒	☐	A description of all covariates tested
☒	☐	A description of any assumptions or corrections, such as tests of normality and adjustment for multiple comparisons
☐	☒	A full description of the statistical parameters including central tendency (e.g. means) or other basic estimates (e.g. regression coefficient) AND variation (e.g. standard deviation) or associated estimates of uncertainty (e.g. confidence intervals)
☒	☐	For null hypothesis testing, the test statistic (e.g. *F*, *t*, *r*) with confidence intervals, effect sizes, degrees of freedom and *P* value noted<br>*Give P values as exact values whenever suitable.*
☒	☐	For Bayesian analysis, information on the choice of priors and Markov chain Monte Carlo settings
☒	☐	For hierarchical and complex designs, identification of the appropriate level for tests and full reporting of outcomes
☒	☐	Estimates of effect sizes (e.g. Cohen's *d*, Pearson's *r*), indicating how they were calculated

Our web collection on statistics for biologists contains articles on many of the points above.

Software and code

Policy information about availability of computer code

Data collection	Flow cytometry data was collected using NovoExpress version 1.2.4. Spectrophotometric assay was performed using a BioTek Synergy Neo2 plate reader and the Gen5 version 3.04 software.
Data analysis	Flow cytometry data was analyzed using the FlowCal Python package version 1.2 (Castillo-Hair et al. ACS Synthetic Biology 5, 774-780, 2016). Analysis of all other data (e.g. calculation of statistics) and plotting was performed using NumPy version 1.17.4, SciPy version 1.3.1, Pandas version 1.0.3, matplotlib version 3.1.1, and DNAplotlib version 1.0 using Python version 3.7.4. Figures were composed using Omnigraffle version 7.15 and Affinity Designer version 1.8.3

For manuscripts utilizing custom algorithms or software that are central to the research but not yet described in published literature, software must be made available to editors and reviewers. We strongly encourage code deposition in a community repository (e.g. GitHub). See the Nature Research guidelines for submitting code & software for further information.

Data

Policy information about availability of data

All manuscripts must include a data availability statement. This statement should provide the following information, where applicable:

- Accession codes, unique identifiers, or web links for publicly available datasets
- A list of figures that have associated raw data
- A description of any restrictions on data availability

Python scripts for simulating the ODE models of the direct and multi-level controllers be found in Supplementary Data 1. Annotated sequences for all plasmids in GenBank format are available in Supplementary Data 2. Source data are provided with this paper. Flow cytometry data is available at: https://osf.io/wm9cq/

Field-specific reporting

Please select the one below that is the best fit for your research. If you are not sure, read the appropriate sections before making your selection.

☒ Life sciences ☐ Behavioural & social sciences ☐ Ecological, evolutionary & environmental sciences

For a reference copy of the document with all sections, see nature.com/documents/nr-reporting-summary-flat.pdf

Life sciences study design

All studies must disclose on these points even when the disclosure is negative.

Sample size	Sample sizes were chosen to match typical practices in the field (e.g. a million cell measured per sample using flow cytometry). Three independent biological replicates were performed for each experiment.
Data exclusions	No data were excluded from the analyses.
Replication	Three biological replicates were preformed for all samples assessed and in all cases good concordance was found between measurements. Average (mean) values are given for all measurements.
Randomization	In each experimental group, the sample was inoculated from a single randomly chosen colony.
Blinding	Blinding is not relevant for this study because our data are not based on qualitative metrics and binding during group allocations is not relevant as cultures were randomly allocated to experimental groups with no control over which cells would be selected for each. Furthermore, this study involved no human or animal participants.

Reporting for specific materials, systems and methods

We require information from authors about some types of materials, experimental systems and methods used in many studies. Here, indicate whether each material, system or method listed is relevant to your study. If you are not sure if a list item applies to your research, read the appropriate section before selecting a response.

Materials & experimental systems

n/a	Involved in the study	
☒	☐	Antibodies
☒	☐	Eukaryotic cell lines
☒	☐	Palaeontology and archaeology
☒	☐	Animals and other organisms
☒	☐	Human research participants
☒	☐	Clinical data
☒	☐	Dual use research of concern

Methods

n/a	Involved in the study	
☒	☐	ChIP-seq
☐	☒	Flow cytometry
☒	☐	MRI-based neuroimaging

Flow Cytometry

Plots

Confirm that:

☒ The axis labels state the marker and fluorochrome used (e.g. CD4-FITC).

☒ The axis scales are clearly visible. Include numbers along axes only for bottom left plot of group (a 'group' is an analysis of identical markers).

☒ All plots are contour plots with outliers or pseudocolor plots.

☒ A numerical value for number of cells or percentage (with statistics) is provided.

Methodology

Sample preparation	Bacterial cultures in exponential phase growth harboring our genetic devices were diluted 1:10 (10 µL into 90 µL) in phosphate-buffered saline (PBS) containing 2 mg/mL kanamycin to halt translation and incubated at room temperature for 1 hour to allow for maturation of the GFP before performing flow cytometry.
Instrument	Acea Biosciences NovoCyte 3000 flow cytometer equipped with a NovoSampler.
Software	Data was analyzed using the FlowCal Python package version 1.2 (Castillo-Hair et al. ACS Synthetic Biology 5, 774-780, 2016).

Cell population abundance	At least 1,000,000 cell measurements were taken for each sample.
Gating strategy	Automated gating was performed by FlowCal version 1.2 using the density2d function with parameters: channels = [‘FSC-A’, ‘SSC-A’], bins = 1024, gate_fraction = 0.5, xscale = ‘logicle’, yscale = ‘logicle’, and sigma = 10.0.

☒ Tick this box to confirm that a figure exemplifying the gating strategy is provided in the Supplementary Information.
