# Supplementary material for: Harnessing the central dogma for stringent multi-level control of gene expression
Source: Nat Commun. 2021 Mar 19;12:1738. doi: 10.1038/s41467-021-21995-7 (PMC7979795; doi:10.1038/s41467-021-21995-7)
Supplement: Supplementary file 5 — Description of Additional Supplementary Files [file 41467_2021_21995_MOESM5_ESM.pdf]

Title: Supplementary Data 1:
Description: Simulation scripts for ODE models of the direct and multi-level controllers.

Title: Supplementary Data 2:
Description: Annotated sequences for all controller plasmids
